# Antimicrobial resistance and genetic diversity of *Klebsiella pneumoniae* strains from different clinical sources in horses

**DOI:** 10.3389/fmicb.2023.1334555

**Published:** 2024-01-11

**Authors:** Francois Gravey, Corinne Sévin, Sophie Castagnet, Nathalie Foucher, Karine Maillard, Jackie Tapprest, Albertine Léon, Bénédicte Langlois, Simon Le Hello, Sandrine Petry

**Affiliations:** ^1^Department of Infectious Agents, Bacteriology, Université de Caen Normandie, Université de Rouen Normandie, INSERM, DYNAMICURE UMR1311, CHU Caen, Caen, France; ^2^Department of Infectious Agents, Bacteriology, CHU Caen, Caen, France; ^3^ANSES, Normandy Laboratory for Animal Health, Physiopathology and Epidemiology of Equine Diseases Unit, Goustranville, France; ^4^Research Department, LABÉO, Caen, France

**Keywords:** *Klebsiella pneumoniae*, horse, K-antigen and O-antigen, MLST, multidrug resistance, whole genome sequencing

## Abstract

**Introduction:**

*Klebsiella pneumoniae* is a major cause of infections and reproductive disorders among horses, ranked in recent French studies as the sixth most frequently isolated bacterial pathogen in equine clinical samples. The proportion of multidrug-resistant (MDR) *K. pneumoniae* is therefore significant in a context where MDR *K. pneumoniae* strains are considered a major global concern by the World Health Organization.

**Methods:**

In this study, we used a genomic approach to characterize a population of 119 equine *K. pneumoniae* strains collected by two laboratories specialized in animal health in Normandy (France). We describe the main antibiotic resistance profiles and acquired resistance genes, and specify the proportion of virulence-encoding genes carried by these strains. The originality of our panel of strains lies in the broad collection period covered, ranging from 1996 to 2020, and the variety of sample sources: necropsies, suspected bacterial infections (e.g., genital, wound, allantochorion, and umbilical artery samples), and contagious equine metritis analyses.

**Results:**

Our results reveal a remarkable level of genomic diversity among the strains studied and we report the presence of 39% MDR and 9% hypervirulent strains (including 5% that are both MDR and hypervirulent).

**Discussion:**

These findings clearly emphasize the importance of improving the surveillance of *K. pneumoniae* in routine equine diagnostic tests to detect high-risk MDR-hypervirulent *Klebsiella pneumoniae* strains. The circulation of these worrisome strains reveals that they are not being detected by the simple K1, K2, and K5 serotype approach currently implemented in the French horse-breeding sector.

## Introduction

*Klebsiella pneumoniae* is ubiquitously isolated from humans, animals, and environmental samples and is the common cause of various infections.

In humans, *K. pneumoniae* is the third most common cause of healthcare-associated infections such as bacteremia, ventilator-associated pneumonia, and urinary tract infections. It has also emerged as an agent of severe community-acquired infections presenting as pyogenic liver abscesses, meningitis, and fasciitis (Holt et al., 2015). These two clinical presentations have been associated with two distinct evolutionary *K. pneumoniae* populations: one of them is multidrug-resistant (MDR), including to carbapenem, and causes nosocomial infections in immunocompromised patients; the other is a mainly wild hypervirulent type (i.e., producing only its chromosomal penicillinase) that causes severe infections in individuals from the community, who are often healthy. MDR *K. pneumoniae* isolates, which produce extended-spectrum β-lactamases (ESBLs) and/or carbapenemases belong to particular clones (Wyres et al., 2020). Thus, the majority of carbapenemase-producing *K. pneumoniae* worldwide belong to clonal group (CG) CG258 (including ST258, ST11, ST340, ST437, and ST512). Several other CGs are also globally distributed and associated with MDR *K. pneumoniae* (Dong et al., 2022). Invasive community-acquired isolates, usually named hypervirulent *K. pneumoniae* were initially described in Asian studies in the 1980s but are increasingly reported worldwide. These isolates differ in clonal background from MDR isolates and appear to predominantly belong to capsular serotypes K1 in CG23 and K2 in other unrelated CGs (Russo and Marr, 2019; Wyres et al., 2020). The hypervirulent *K. pneumoniae* virulomes are also well documented, in particular the importance of the pLVPK-like virulence plasmid and/or pathogenicity island in mucoid phenotype expression. Worryingly, the evolutionary convergence of MDR and hypervirulent *K. pneumoniae* high-risk clones raises serious therapeutic challenges. Its recent global spread has been associated with a diverse genetic background (Arcari and Carattoli, 2023).

In animals, most clinical manifestations of *K. pneumoniae* infections concern the urinary and respiratory tracts, which could lead to sepsis. In addition, among horses, *K. pneumoniae* is a major cause of abortion (Laugier et al., 2011; Akter et al., 2021) or metritis (Platt and Atherton, 1976), and may occasionally cause infertility issues in inseminated mares (Malaluang et al., 2021). Three recent French retrospective studies showed that *K. pneumoniae* is the sixth most isolated bacterial pathogen of equine clinical samples (Duchesne et al., 2019; Bourély et al., 2020; Léon et al., 2020), with an annual frequency of MDR strains ranging from 11.7 to 51.5% over the 2006–2019 period (Duchesne et al., 2019; Léon et al., 2020). A recent Israeli case control study on 3GC-resistant *Enterobacterales* infections in hospitalized horses and donkeys showed that the *Klebsiella* spp. were the most common 3GC-resistant *Enterobacterales* detected (Shnaiderman-Torban et al., 2021). However, it remains difficult to detect high-risk clonal lineages of *K. pneumoniae* through epidemiological and genomic analyses in horses (e.g., Trigo da Roza et al., 2019; Loncaric et al., 2020; Shnaiderman-Torban et al., 2021). A recent molecular study compared genomes from hypervirulent CG23 *K. pneumoniae* strains of both human and horse origins (Lam et al., 2018), but very little information is currently available regarding the hypervirulence of *K. pneumoniae* in horses.

The objectives of the present study were to (i) characterize by a genomic approach a population of 119 equine *K. pneumoniae* strains collected by two laboratories specialized in animal health in Normandy (France), and responsible for various manifestations of infection, (ii) describe the main antibiotic resistance profiles and acquired resistance genes, and (iii) describe the proportion of virulence-encoding genes carried by these strains. In addition to the numerous strains analyzed, the originality of this equine *K. pneumoniae* population lies in the broad collection period covered, ranging from 1996 to 2020, and the variety of sample sources: necropsies, suspected bacterial infections (e.g., genital, wound, allantochorion, and umbilical artery samples), and contagious equine metritis (CEM) analyses.

## Materials and methods

### Bacterial isolates

In all, 119 French *K. pneumoniae* strains were investigated in the present study, all from horses (Supplementary material). Between 1996 and 2020, the ANSES Normandy Laboratory for Animal Health isolated 118 *K. pneumoniae* strains from 117 necropsies and one analysis of the allantochorion from an abortion (2.6% of total necropsies performed with an aerobic bacteriological search between 1996 and 2020). Among the strains investigated, 23 were excluded from the study: ten were no longer cultivable and 13 were in fact not members of the *K. pneumoniae* species according to the *in-silico* analyses. The ANSES laboratory thus supplied 94 strains considered here as “necropsy-associated strains.” The microbiology diagnostic unit of LABÉO laboratory provided a total of 25 strains isolated between 2011 and 2020, considered here as “non-necropsy-associated strains.” These strains were isolated from genital samples (*n* = 20), which included 12 CEM cultures, wounds (*n* = 4), and the umbilical artery (*n* = 1). They were selected based on their antibiotic susceptibility profile and genital origin, which was the major source of sample origin for *K. pneumoniae* at LABÉO (Duchesne et al., 2019; Léon et al., 2020). ANSES’s strains were stored using CryoBeads™ (bioMérieux) at a temperature ≤ −65°C and LABÉO’s strains were conserved at-80°C on brain heart infusion broth with 10% glycerol. Before phenotypic analyses and genome sequencing, all the strains were cultivated on ready-to-use 5% sheep blood agar (bioMérieux) incubated at 35 ± 2°C for 24 h.

### Phenotypic antimicrobial susceptibility testing

Antimicrobial susceptibility testing was performed using the disk diffusion method on Mueller-Hinton agar plates according to the European Committee on Antimicrobial Susceptibility Testing (EUCAST) guideline.^1^ The panel of 35 antibiotics (BioRad) tested included amikacin, amoxicillin-clavulanate, aztreonam, cefalexin, cefamandole, cefepime, cefoxitin, ceftazidime, ceftiofur, ceftolozane-tazobactam, ceftriaxone, chloramphenicol, ciprofloxacin, ertapenem, fosfomycin, gentamicin, imipenem, kanamycin, levofloxacin, marbofloxacin, mecillinam, meropenem, nalidixic acid, netilmicin, norfloxacin, piperacillin-tazobactam, spectinomycin, streptomycin, temocillin, tetracycline, ticarcillin-clavulanate, tigecycline, tobramycin, trimethoprim and trimethoprim-sulfamethoxazole. They were then interpreted as susceptible, intermediate, or resistant using the breakpoints for *Enterobacterales* available in the 2021 veterinary recommandations of the Comité de l’Antibiogramme de la Société Française de Microbiologie (CASFM, 2021) or, failing that, those available for *Enterobacterales* strains of human origin (CASFM, 2013; CASFM/EUCAST, 2021) (Supplementary material). Of note, strains that were categorized as intermediate were considered as resistant for further descriptive and comparative statistical analyses.

MDR strains have been defined as antimicrobial resistance shown by a species of microorganism to at least one antimicrobial drug in three or more antimicrobial categories as previously described (Magiorakos et al., 2012). The MDR classification complied with the 35 antibiotics.

### K125 PCR

A multiplex PCR was performed using targets within the serotype-specific region of the capsular polysaccharide synthesis gene cluster of serotypes K1, K2, and K5 using primers described previously (Turton et al., 2008). Before the amplification, each strain was suspended in 500 μL of PBS solution compliant with McFarland 0.5 and heated to 95°C for 10 min then centrifuged at 1800 g for 1 min. PCR assays were performed using the Multiplex PCR kit (Qiagen) according to the manufacturer’s recommendation. In brief, after an initial 15-min step at 95°C, the analyses were run on a Verity thermal cycler (Applied Biosystems, Thermo Fisher). There were 45 cycles of denaturation at 94°C for 30 s, primer annealing at 60°C for 90 s and elongation at 72°C for 90 s, completed by a final 10-min step at 72°C. To ensure the validity of the assays, positive and negative controls were run in parallel. The length of the amplicons (1,283, 641 and 276 bp for the K1, K2, and K5 capsular polysaccharide synthesis gene, respectively) was verified using the Qiaxcel Advanced System (Qiagen).

### Whole genome sequencing

Isolates were subcultured for 6 h in Luria-Bertani broth at 35 ± 2°C, then 1 mL was centrifuged for 10 min at 4,000 rpm. The pellet was then resuspended in 100 μL PBS 1X. Cells were lysed using MagNA Pure Lysis Buffer (Roche) as well as Proteinase K recombinant PCR Grade (Roche). DNA was extracted from each isolate using the MagNA Pure system (Roche).

Dual-indexed Illumina sequencing libraries were constructed from each sample using the Nextera XT DNA library preparation kit (Illumina), pooled, then sequenced on the Illumina NextSeq 500 platform (Plateforme de Microbiologie Mutualisée P2M, Institut Pasteur, Paris, France). Sequencing was performed according to the manufacturer’s instructions using a 2 × 150-bp paired-end configuration with a minimum coverage of 30x. Quality control was carried out on the raw reads using FastQC (Andrews, 2010) and MultiQC (Ewels et al., 2016). Adapters and reads with a median Phred score lower than 25 and/or shorter than 70 pb were removed from the fastq files using fqCleanER.^2^ Genomes were assembled using the SPAdes Genome Assembler v.3.12.0 with parameters recommended for 150 pb paired-end reads (k-mer lengths of 21, 33, 55, 77) (Bankevich et al., 2012). The quality of the assemblies was assessed in Quast (Gurevich et al., 2013). Genomes and associated metadata are available on the *Klebsiella* BIGSdb-Pasteur database^3^ in the “Klebsequi-projet” public project. Genomes are also available on the NCBI database using the Bioproject number PRJNA1054041.

### *In silico* WGS analysis

Several *in silico* analyses were performed for each genome: (i) species were checked using the rMLST software (Jolley et al., 2012); (ii) sequence types (STs) were attributed according to the multi locus sequence typing (MLST) scheme previously described using the BIGSdb-Kp database (Diancourt et al., 2005). Unknown or new STs were submitted to the BIGSdb-Kp database for curation (see footnote 3). A Venn diagram was plotted to illustrate overlaps of the source types/hosts (i.e., human, horse, animal other than horse, environment, and food) for STs identified in this study and already present in the BIGSdb-Kp database; (iii) the lipopolysaccharide O-antigen was characterized by the nucleotide sequence of the *wzm* gene using the Kaptive command line tool^5^ (Follador et al., 2016; Wick et al., 2018) with “Good” as the confidence threshold (any result below the threshold was “not assigned”); (iv) the capsular polysaccharide K-antigen was determined by the nucleotide sequence of the *wzi* gene (Brisse et al., 2013) using the BIGSdb-Kp database; (v) a search was conducted for all the virulence-encoding genes into the BIGSdb-Kp database with a 90% identity threshold using the KMA software (Bialek-Davenet et al., 2014; Clausen et al., 2018); (vi) acquired antibiotic resistance genes and chromosomal mutations were determined using both ResFinder and rgi databases with a 90% identity and coverage threshold, respectively (Zankari et al., 2012; Alcock et al., 2020); (vii) the PlasmidFinder tool (Carattoli et al., 2014) was used to look for families of plasmids carried by the strains, including the pK2044 and pLVPK-like virulence plasmids. The tool plasmidfinder look for replicon sequences from plasmid among fastq reads or assembled sequences. Using small replicon sequences allow the identification of plasmids sequences using short-read sequencing data. Hypervirulent strains were defined based on the presence of several biomarkers according to Russo et al. (2018) (e.g., *peg-344*, *iroB*, *iucA*, *rmpA*, and *rpmA2*).

To further distinguish the isolates, cgMLST analysis using the chewBBACA (Silva et al., 2018) “*K. pneumoniae sensu lato* cgMLST” version 1.0 scheme with a total of 2,358 genes^6^ was performed using all the genomes. Only genes for which alleles were found in all isolates were kept for analysis. A minimum spanning tree (MST) was constructed using GrapeTree (Zhou et al., 2018). Sublineage and clonal groups (CGs) were attributed for each strain using the core genome MLST scheme based on 629 genes previously described (Hennart et al., 2022).

### Statistical analysis

Statistical analysis was performed using RStudio version 2022.12.0. Association between category variables and the MDR status of strains was tested with Pearson’s Chi-squared test and Fisher’s exact test where appropriate (*n* < 5). We did not impute unknown values and defined statistical significance as a *p*-value <0.05.

## Results

### Description of the equine *Klebsiella pneumoniae* population

The 94 (79.0%) necropsy-associated strains and 25 (21.0%) non-necropsy-associated strains studied were from 119 horses located in nine French regions, though predominantly from Normandy (79.8%) in accordance with the location of the two laboratories that isolated the strains. At least eight horse breeds were represented, with an overdistribution of Trotter or French Trotter (39.5%) and Thoroughbred (33.6%) mostly skewed by the necropsy recruitment. The sex (male: female) ratio of 0.63 is skewed mostly due to the dominance of genital origins for the non-necropsy-associated strains, and stands at 0.82 when only necropsy-associated strains are taken into account. Age ranged from fetus to 22 years old, and year of strain isolation ranged from 1996 to 2020: (i) necropsy-associated strains were from 18 fetuses (median, 8.5 months of gestation; range, 3.75–9.5 months of gestation), 63 foals (median, 1 month; range, 0–17 months), and 13 adult horses (median, 8 years; range, 3–22 years), and were isolated from 1996 to 2020 (median, 2007); (ii) non-necropsy-associated strains were from seven unknown and 18 adult horses (median, 11 years; range, 4–20 years), and were isolated from 2011 to 2020 (median, 2019). The clinical source of strain isolations was varied (Supplementary material) but it could be grouped into sepsis (21.8%), digestive (20.2%), genital (17.6%), abortion (15.1%), respiratory (13.5%, including *Rhodococcus equi* infections), cutaneous (3.4%), and other (8.4%, including fractures, anoxia/asphyxia, viral/parasitic infections, umbilical artery, cardiac tamponade, hepatic tear, cervical trauma, myopathy, and unknown). Furthermore, co-infections with other bacteria were found in 89.9% of cases (Supplementary material); the most frequent co-infections were with *Escherichia coli* (37%), *Staphylococcus xylosus* (15%) and *Streptococcus equi* subsp. *zooepidemicus* (14%), followed by, e.g., *Streptococcus* other than *S. zooepidemicus* (8%), *Enterococcus* spp. (8%), *Rhodococcus equi* (7%), *Clostridium difficile* (4%), *Pseudomonas aeruginosa* (4%) (Supplementary material).

For the 94 necropsy-associated strains, lesions related to the cause of death were observed in 69.1% of cases, while lesions unrelated to the cause of death (12.8%) and absence of lesions (17.0%) were also observed.

### Antimicrobial susceptibilities

All 119 strains were resistant to at least one class of antibiotics tested, with a median of 1/35 antibiotics categorized as resistant per strain, and 46 (38.7%) presented an MDR phenotype according to the definition mentioned above, with a median of 17/35 molecules categorized resistant (Supplementary material). The most frequent resistant classes were ß-lactams, aminoglycosides and tetracyclines, followed by pyrimidines, phenicols, quinolones, and phosphoric acid (Table 1). The *in silico* WGS analysis showed the presence of three to 20 resistance genes per strain, with a median of four when all strains were considered and a median of 14 when only MDR strains were considered (Supplementary material).

**Table 1:** Antimicrobial susceptibility profiles of the 119 *K. pneumoniae* strains studied.

Antibiotic class	Family	Antibiotic	Susceptible [*n* (%)]	Resistant [*n* (%)]
β-lactams	Penicillin	MEC	114 (95.8)	5 (4.2)
		TEM	0 (0)	119 (100)
	Penicillin/β-lactamase inhibitor	AMC	91 (76.5)	28 (23.5)
		TIL	90 (75.6)	29 (24.4)
		PIT	112 (94.1)	7 (5.9)
	1GC	CLE	88 (73.9)	31 (26.1)
	2GC	CMA	88 (73.9)	31 (26.1)
		CXI	109 (91.6)	10 (8.4)
	3GC	CTZ	89 (74.8)	30 (25.2)
		CTR	90 (75.6)	29 (24.4)
		CTF	96 (80.7)	23 (19.3)
	4GC	CEP	98 (82.4)	21 (17.6)
	Cephalosporin/β-lactamase inhibitor	CTT	117 (98.3)	2 (1.7)
	Carbapenem	ERT	118 (99.2)	1 (0.8)
		IMI	119 (100)	0 (0)
		MER	119 (100)	0 (0)
	Monobactam	AZT	97 (81.5)	22 (18.5)
Aminoglycosides		STR	73 (61.3)	46 (38.7)
		GEN	92 (77.3)	27 (22.7)
		NET	94 (79.0)	25 (21.0)
		KAN	94 (79.0)	25 (21.0)
		TOB	96 (80.7)	23 (19.3)
		SPE	100 (84.0)	19 (16.0)
		AMI	118 (99.2)	1 (0.8)
Quinolones	Quinolone	NAL	105 (88.2)	14 (11.8)
	Fluoroquinolone	CIP	97 (81.5)	22 (18.5)
		LEV	105 (88.2)	14 (11.8)
		NOR	108 (90.8)	11 (9.2)
		MAR	111 (93.3)	8 (6.7)
Pyrimidines		TRI	86 (72.3)	33 (27.7)
		TRS	87 (73.1)	32 (26.9)
Phosphoric acid		FOS	116 (97.5)	3 (2.5)
Phenicol		CHL	98 (82.4)	21 (17.6)
Tetracyclines		TET	81 (68.1)	38 (31.9)
		TIG	118 (99.2)	1 (0.8)

MEC, mecillinam; TEM, temocillin; AMC, amoxicillin-clavulanate; TIL, ticarcillin-clavulanate; PIT, piperacillin-tazobactam; CLE, cefalexin; CMA, cefamandole; CXI, cefoxitin; CTZ, ceftazidime; CTR, ceftriaxone; CTF, ceftiofur; CEP, cefepime; CTT, ceftolozane-tazobactam; ERT, ertapenem; IMI, imipenem; MER, meropenem; AZT, aztreonam; STR, streptomycin; GEN, gentamicin; NET, netilmicin; KAN, kanamycin; TOB, tobramycin; SPE, spectinomycin; AMI, amikacin; NAL, nalidixic acid; CIP, ciprofloxacin; LEV, levofloxacin; NOR, norfloxacin; MAR, marbofloxacin; TRI, trimethoprim; TRS, trimethoprim-sulfamethoxazole; FOS, fosfomycin; CHL, chloramphenicol; TET, tetracycline; TIG, tigecycline.

Among ß-lactams, 83 strains (69.7%) presented a single resistance to temocillin, and they only produced the chromosomal narrow spectrum ß-lactamase SHV-1 or its variants. Thirty-one strains (26.1%) were resistant to at least one 3/4GC tested. Ceftazidime was the most frequent hydrolyzed 3/4GC, followed by ceftriaxone, ceftiofur, and cefepime (respectively 25.2, 24.4, 19.3, and 17.6%) (Table 1). Mechanisms involved other than the 3/4GC resistance phenotype were mainly ESBL production (*n* = 22, 71.0%), followed by plasmid-mediated AmpC cephalosporinase (*n* = 9, 29.0%). Among the plasmid-mediated AmpC cephalosporinase, the *bla*_CMY-2_ gene was the most frequently found (*n* = 7), followed by *bla*_DHA-1_ (*n* = 2). Regarding the ESBL-encoding genes, *bla*_CTX-M-15_ (*n* = 14) was the most frequently encountered gene, followed by *bla*_CTX-M-2_ (n = 3), *bla*_CTX-M-3_ (*n* = 2), and *bla*_SHV-12_ (*n* = 2), respectively (Supplementary material). Only one strain (strain 009) was resistant to ertapenem and none to imipenem or meropenem; this strain did not produce any carbapenemase, but the production of DHA-1 could lead to low-level resistance to ertapenem (Jacoby, 2009), especially if the cephalosporinase is associated with efflux pump overexpression and/or porin loss (Table 1; Supplementary material). Finally, only two strains were resistant to ceftolozan-tazobactam: one produced DHA-1 (strain 124) and the other ESBL, CTX-M-15, and SHV-12 (strain 131) (Table 1; Supplementary material).

Regarding the aminoglycoside class, streptomycin, gentamicin, kanamycin, and netilmicin were the most frequent ineffective antibiotics with the following resistance rates: 38.7, 22.7, 21.0, and 21.0%, respectively (Table 1). In contrast, only one strain (strain 126) was considered resistant to amikacin. Twenty aminoglycoside resistance genes were recovered from 48/119 strains, the most prevalent being *aph(6)-Id* (*n* = 39), *aph(3″)-Ib* (*n* = 32), and *aph(3′)-Ia* (*n* = 17) (Supplementary material).

One third of the strains were resistant to tetracyclines due to the presence of genes coding for a major facilitator superfamily (MFS) antibiotic efflux pump, respectively, *tet(A)* (*n* = 31), *tet(B)* (*n* = 5), *tet(D)* (*n* = 3), and *tet(C)* (*n* = 1) (Supplementary material). One resistant strain (strain 048) did not harbor a *tet* family gene but had a mutation in the *kpnF* gene which codes a subunit of a two-component system, KnpEF, involved in broad-spectrum antimicrobial resistance (Srinivasan and Rajamohan, 2013).

There was substantial resistance to pyrimidines: 27.8% to trimethoprim and 26.9% to trimethoprim-sulfamethoxazole (Table 1). Thirteen strains had a *sul* gene alone (*sul1*, *n* = 6; *sul2*, *n* = 7) with no phenotypic consequences for cotrimoxazole. In contrast, 32 pyrimidine-resistant strains had *sul* genes associated with dihydrofolate reductase encoding *dfrA*. The most frequent *dfrA* genes were *dfrA14* (*n* = 12), *dfrA12* (*n* = 10), *dfrA1* (*n* = 6), and *dfrA27* (*n* = 4), respectively (Supplementary material).

Concerning quinolone and fluoroquinolone, the most frequent resistant molecules were ciprofloxacin, nalidixic acid, and levofloxacin, respectively (18.5, 11.8, and 11.8%) (Table 1). Several mechanisms were involved; some strains had the gyrA-83I and parC-80I mutations inside the quinolone resistance-determining region leading to a complete resistance class (*n* = 4), while others had quinolone resistance genes (*qnr*), mainly *qnrS1* (*n* = 11), *qnrB1* (*n* = 8), and *qnrB4* (*n* = 2) (Supplementary material). Moreover, several efflux pump systems were found to be involved, particularly among nalidixic acid-resistant strains: A repressor of the MdtEF pump called CRP was mutated, as was subunit KpnG of the KpnHH efflux pump (Supplementary material). Marbofloxacin was the most active fluoroquinolone tested, 93.3% of the strains being susceptible to it (Table 1).

Twenty-one strains (17.6%) were resistant to chloramphenicol. The FloR exporter was the most frequent chloramphenicol resistance mechanism (*n* = 12), followed by the presence of chloramphenicol acetyltransferase *cat* genes (*n* = 7) and dysregulations among RND pumps (*n* = 4): Mutations in *marA* and *acrB* (Supplementary material).

Aside from two strains (strains 35 and 63), *fosA* genes were found in the genome of all the strains (98.3%). The most frequent was *fosA6* (*n* = 85), followed by *fosA5* (*n* = 26), *fosA7* (*n* = 5), and *fosA3* (*n* = 1) (Supplementary material). Despite the quasi omnipresence of the *fosA* gene in the genomes, only three strains were resistant to fosfomycin (Table 1). Among tetracyclines, 38 strains (31.9%) were resistant to tetracycline but only one was found to be resistant to tigecycline, which was a very active molecule (Table 1). Even if the correlation between genotype and phenotype was not performed due to natural resistance for macrolides and rifampicin, there were many resistance-encoding genes. Regarding the macrolide, lincosamide and streptogramin molecules, gene-coding macrolide phosphotransferases were predominant: *mph(A)* (*n* = 21), *mph(E)* (*n* = 2), and *mph(B)* (*n* = 1), followed by *ere(A)* and *msr(E)* (*n* = 2 for both). On the rifampicin side, two different types of rifampin ADP-ribosyltransferase were found among the genomes, *arr-3* (*n* = 12) and *arr-8* (*n* = 1). Finally, one *mcr-9* gene conferring resistance to colistin (molecule not tested in the present study) was found in strain 132 (Supplementary material).

### Antimicrobial susceptibilities and MDR prevalence in necropsy-associated strains

**Figure 1:**
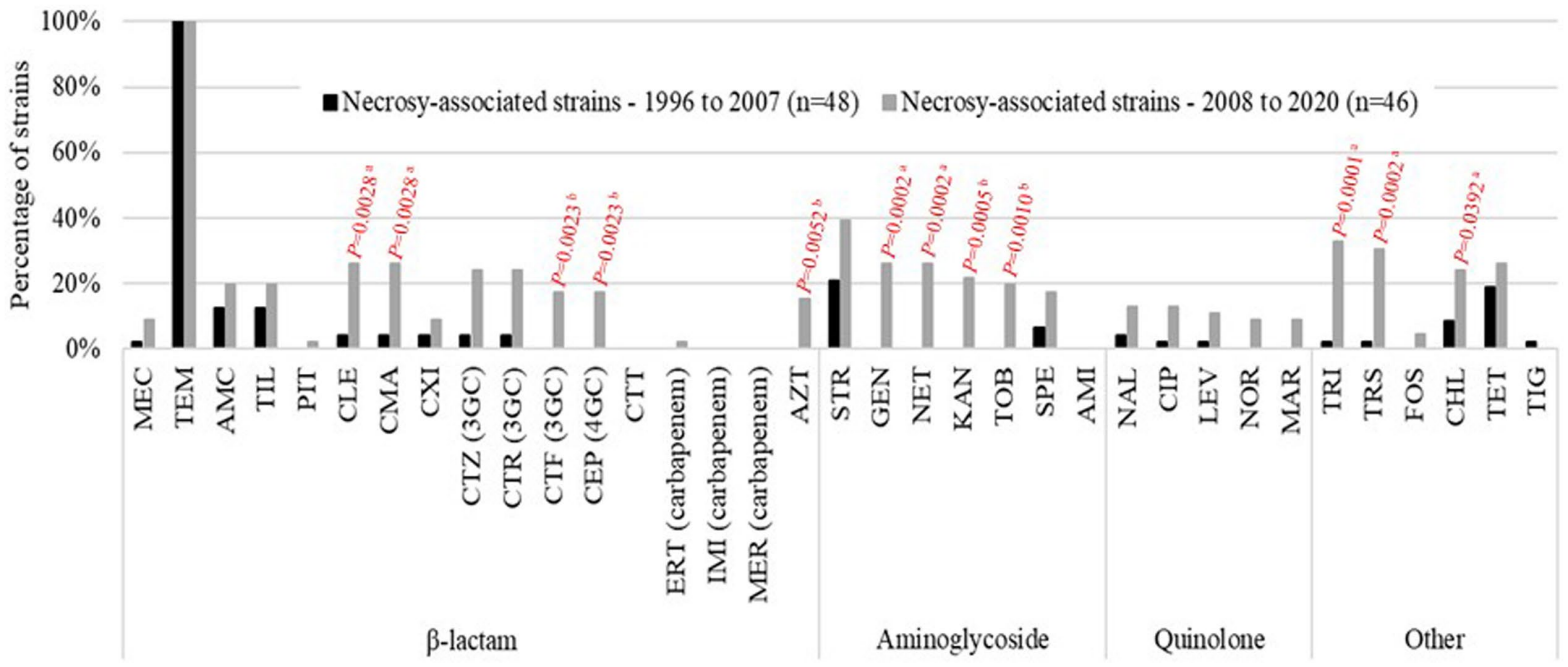
Percentage of resistant results for the equine necropsy-associated *K. pneumoniae* strains studied. Comparison between the 1996–2007 period (*n* = 48; black bars) and the 2008–2020 period (*n* = 46; gray bars). Statistically significant differences are indicated in red (^a^Pearson’s Chi-squared test; ^b^Fisher’s exact test).

The focus on the 94 necropsy-associated strains showed lower resistance to those observed when all 119 strains are considered. However, this difference cannot be taken into consideration as almost all non-necropsy-associated strains were selected based on their antimicrobial drug profile. It is the same way for the lower MDR proportion observed among the necropsy-associated strains (*n* = 27, 28.7%) compared to the MDR proportion in all 119 strains studied (*n* = 46, 38.7%). Interestingly, a large difference was observed between the 1996–2007 and 2008–2020 periods of necropsy-associated strain isolations (Figure 1). The 2008–2020 period showed that resistances had increased by 2.2 to 30.5% for 29/35 antibiotics compared with the 1996–2007 period; only amikacin, ceftolozane-tazobactam, imipenem, meropenem, and temocillin showed equivalent resistances, and tigecycline resistance had decreased by 2.1%. The increased resistances during the 2008–2020 period were statistically significant for 12 antibiotics, with *p*-values from 0.0001 to 0.0392 (represented in red in Figure 1). This difference was also observed considering the MDR proportion between the 1996–2007 period (nine MDR/48, 18.8%) and 2008–2020 period (18 MDR/46, 39.1%) of necropsy-associated strain isolations. The bigger MDR proportion for the 2008–2020 period than the 1996–2007 period was statistically significant (Table 2). The MDR distribution in the necropsy-associated strains was also analyzed considering the information on horses (sex, age and breed) and clinical presentations (Table 2). Thus, statistically significant differences were observed, such as a higher MDR proportion for categories “foal,” “Thoroughbred,” “digestive,” and “presence of lesions not related to the cause of death,” and a lower MDR proportion for categories “fetus,” “abortion” and “co-infection with *Streptococcus zooepidemicus*.” The lower MDR proportion in category “presence of lesions related to the cause of death” was not statistically significant (Chi^2^, *p* = 0.0539).

**Table 2:** Distribution of MDR and non-MDR necropsy-associated *K. pneumoniae* strains according to clinical presentation and co-infection.

Parameter	MDR (*n* = 27) [*n* (%)]	no MDR (*n* = 67) [*n* (%)]	*p*-value
*Isolation period*			
1996–2007	9 (33.3)	39 (58.2)	**0.0290** ^b^
2008–2020	18 (66.7)	28 (41.8)	**0.0290** ^b^
*Sex* *^a^*			
Female	14 (51.9)	37 (55.2)	0.7112^b^
Male	13 (48.1)	29 (43.3)	0.7112^b^
*Age range*			
Fetus	1 (3.7)	17 (25.4)	**0.0157** ^b^
Foal	23 (85.2)	40 (59.7)	**0.0174** ^b^
Adult	3 (11.1)	10 (14.9)	0.7509^c^
*Breed* *^a^*			
Thoroughbred	13 (48.1)	16 (23.9)	**0.0239** ^b^
Trotter	12 (44.5)	34 (50.7)	0.5359^b^
Saddlebred	2 (7.4)	11 (16.4)	0.3333^c^
Other	0 (0.0)	5 (7.5)	0.3165^c^
*Clinical source*			
Abortion	1 (3.7)	17 (25.4)	**0.0157** ^b^
Digestive	13 (48.2)	11 (16.4)	**0.0014** ^b^
Sepsis	5 (18.5)	21 (31.3)	0.2085^b^
Respiratory	5 (18.5)	11 (16.4)	0.0933^c^
Other	3 (11.1)	7 (10.5)	1.0000^c^
*Lesions observed at the necropsy*			
Absence	5 (18.5)	11 (16.4)	1.0000^c^
Presence not related to the cause of death	7 (25.9)	5 (7.5)	**0.0353** ^c^
Presence related to the cause of death	15 (55.6)	50 (74.6)	0.0539^b^
*Co-bacterial identification* *^a^*			
None	2 (7.4)	1 (1.5)	0.2011^c^
*Actinobacillus equuli*	0 (0.0)	3 (4.5)	0.5537^c^
*Aeromonas* spp.	0 (0.0)	2 (3.0)	1.0000^c^
*Clostridium difficile*	2 (7.4)	3 (4.5)	0.6256 ^c^
*Clostridium perfringens*	0 (0.0)	2 (3.0)	1.0000^c^
*Enterococcus* spp.	3 (11.1)	4 (6.0)	0.3516^c^
*Escherichia coli*	10 (37.0)	28 (41.8)	0.7291^b^
*Proteus vulgaris*	1 (3.7)	0 (0.0)	0.2903^c^
*Pseudomonas aeruginosa*	1 (3.7)	2 (3.0)	1.0000^c^
*Pseudomonas cepacia*	0 (0.0)	2 (3.0)	1.0000^c^
*Rhodococcus equi*	2 (7.4)	6 (9.0)	1.0000^c^
*Salmonella* spp.	1 (3.7)	3 (4.5)	1.0000^c^
*Serratia rubidaea*	0 (0.0)	1 (1.5)	1.0000^c^
*Staphylococcus aureus*	3 (11.1)	1 (1.5)	0.0721^c^
*Staphylococcus xylosus*	4 (14.8)	14 (20.9)	0.4784^b^
*Steptococcus equi*	0 (0.0)	1 (1.5)	1.0000^c^
*Streptococcus equisimilis*	2 (7.4)	4 (6.0)	1.0000^c^
*Streptococcus zooepidemicus*	0 (0.0)	16 (23.9)	**0.0046** ^c^
*Candida famata*	0 (0.0)	1 (1.5)	1.0000^c^

a One unknown data excluded (no MDR strain 114).

b Pearson’s Chi-squared test.

c Fisher’s exact test.

### High diversity of MLST genotypes confirmed by cgMLST

**Figure 2:**
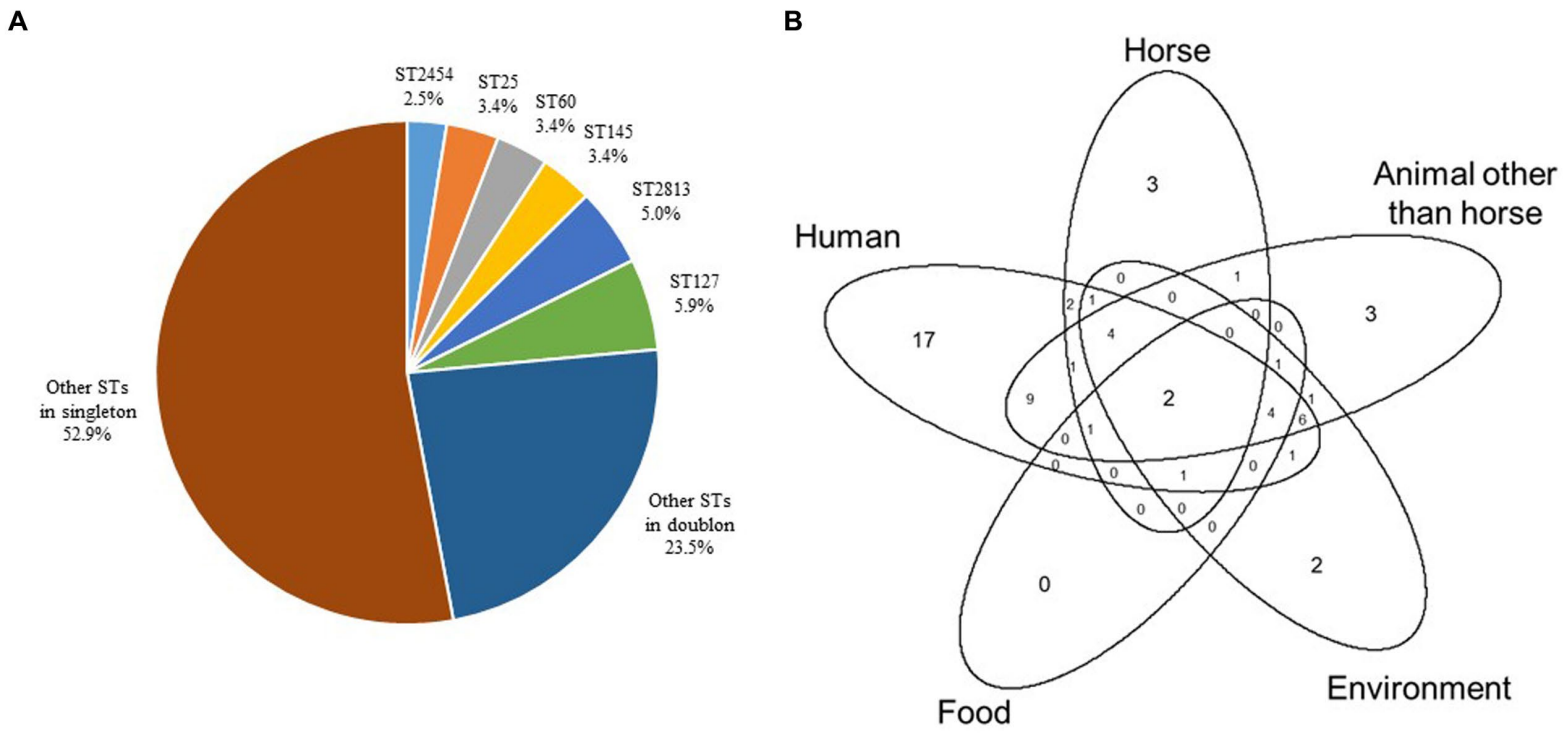
Distribution of ST genotypes in the 119 equine *K. pneumoniae* strains studied. **(A)** Distribution (%) of ST genotypes. **(B)** Venn diagram to illustrate overlaps of the source types/hosts (i.e., human, horse, animal other than horse, environment, and food) of STs identified in this study and already present in the BIGSdb-Kp database (https://bigsdb.pasteur.fr/klebsiella/).

The population studied reveals a remarkable level of genomic diversity since 83 different STs were assigned among the 119 genomes, including 63 singletons. ST127 (*n* = 7), ST2813 (*n* = 6), ST25, ST60, ST145 (*n* = 4 each), and ST2454 (*n* = 3) were the most prevalent STs and concerned nearly a quarter of the strains (Figure 2A, Supplementary material). ST25 (Fisher’s test, *p* = 0.021), ST127 (Fisher’s test, *p* = 0.013) and ST2813 (Fisher’s test, *p* = 0.003) were statistically more frequent in MDR strains. The analysis of the source types/hosts of the STs already present in the BIGSdb-Kp database showed that 49 STs had been previously reported in humans, 33 in animals other than horses, 23 in the environment, 16 in horses (33 STs of equine origin in the BIGSdb-Kp database were not taken into account since they were new STs identified in this study) and nine in food, with multiple overlaps (Supplementary material, Figure 2B).

**Figure 3:**
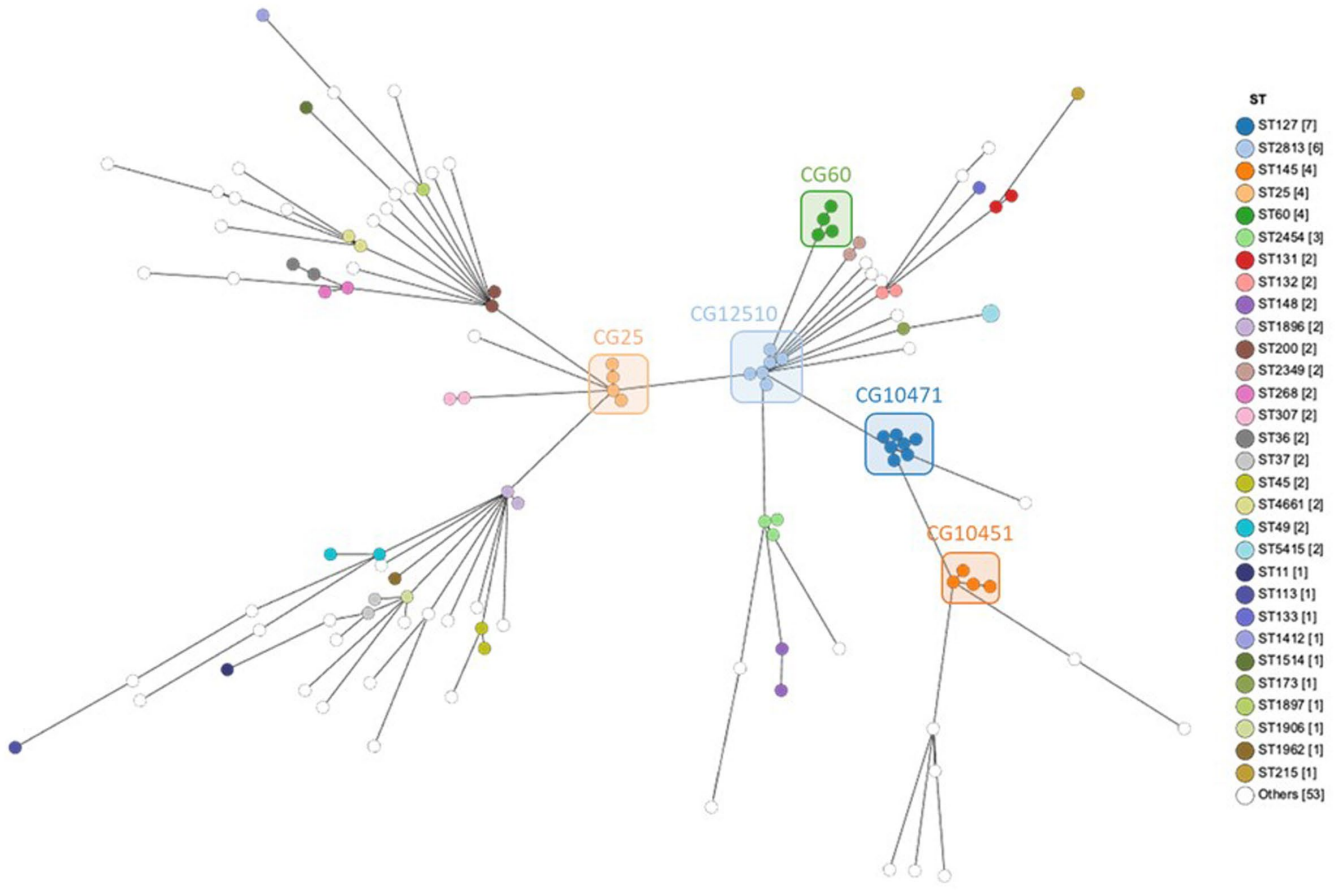
MST showing cgMLST analysis of 119 equine *K. pneumoniae* studied (1809 genes without missing data) according to the ST assignment. Node color indicates the ST assignment. The five most frequent CGs are boxed.

**Figure 4:**
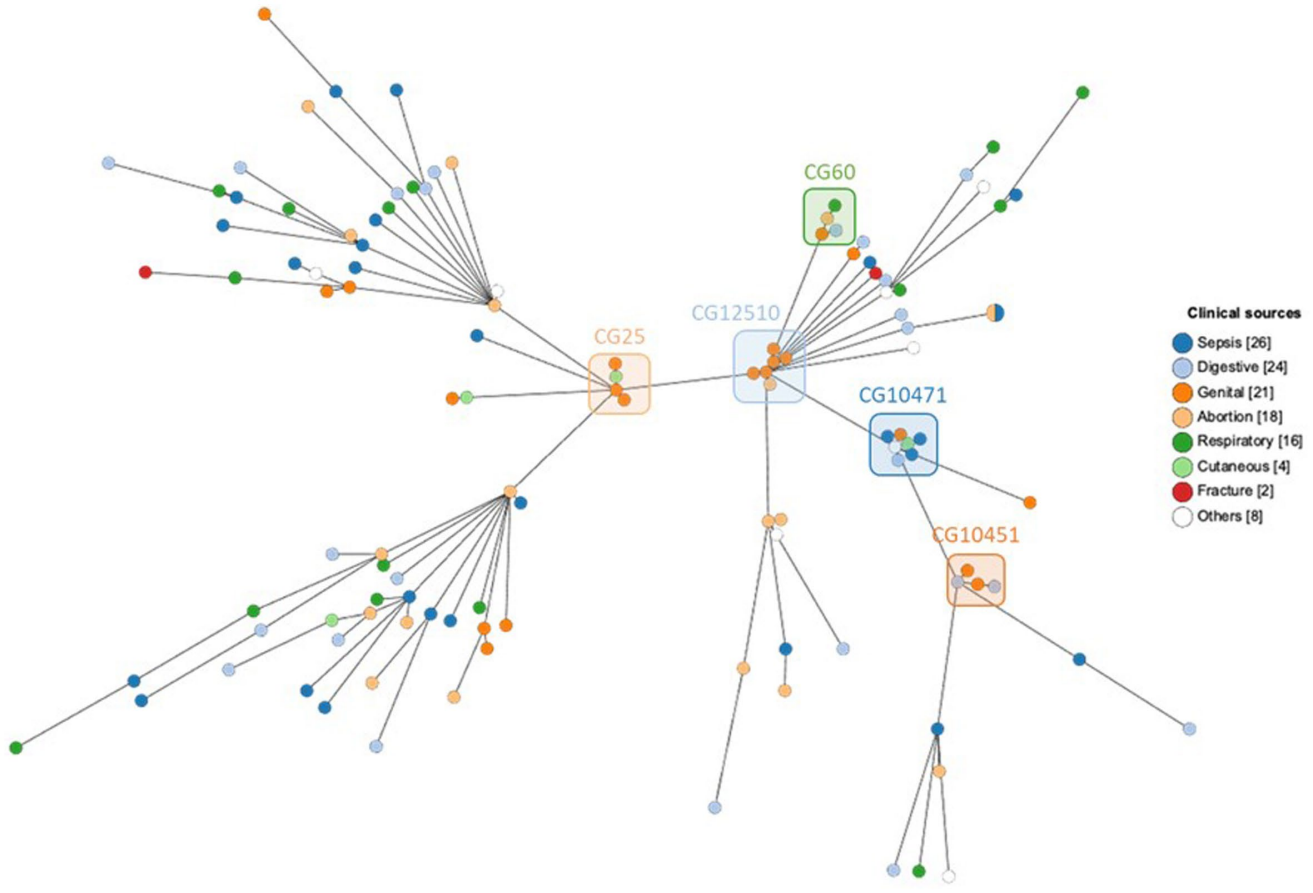
MST showing cgMLST analysis of 119 equine *K. pneumoniae* studied (1809 genes without missing data) according to the clinical sources. Node color indicates the clinical sources. The five most frequent CGs are boxed.

**Figure 5:**
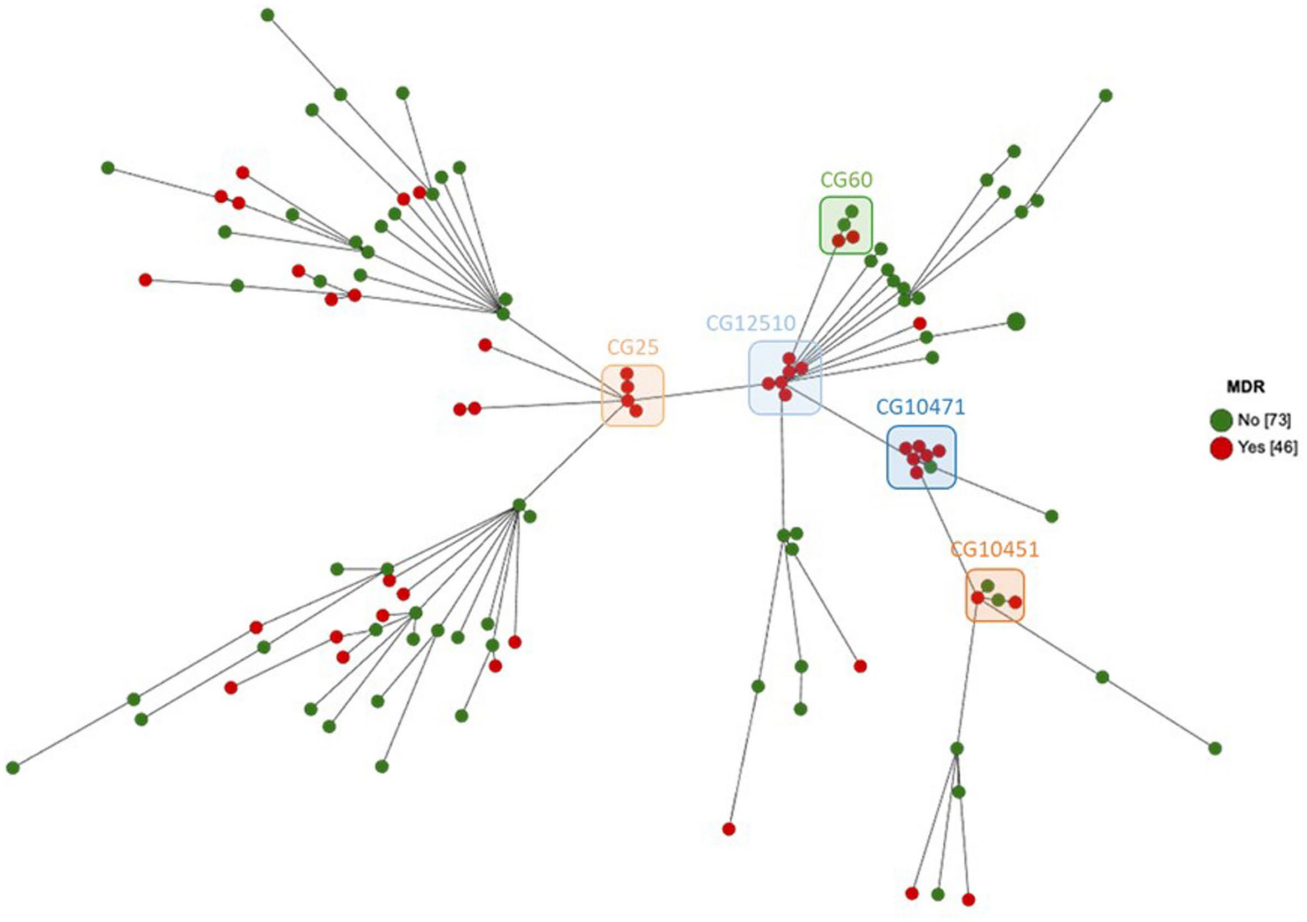
MST showing cgMLST analysis of 119 equine *K. pneumoniae* studied (1809 genes without missing data) according to the MDR phenotype. Node color indicates the MDR phenotype. The five most frequent CGs are boxed.

The genome comparison by cgMLST was carried out on 1809 genes after excluding 549 genes with missing values. In total, 66,402 single alleles were identified with a median of 38 alleles per gene (range 1–87). The median distance between the strains was 1,520 alleles with a maximum distance of 1,580 alleles, reflecting a very diverse population. Only a few strains were much closer to each other, e.g., both strains of ST5415 shared the same 1809 alleles, strains 101 and 102 of ST2454 were distant by only two alleles, all six strains of ST2813 had a maximum distance of seven alleles, strains 82, 83, and 107 had a maximum distance of nine alleles, and all four strains of ST25 had a maximum distance of 11 alleles (Supplementary material). Phylogenetic relations between strains assessed using cgMLST are visualized as MST according to ST distribution (Figure 3), clinical sources (Figure 4), and MDR phenotype (Figure 5). Among the 72 CGs assigned (Supplementary material), the five most frequent CGs were CG10451 (seven ST127 strains from diverse clinical sources, including six that were MDR), CG12510 (six ST2813 and MDR strains from genital and abortion sources), CG25 (four ST25 and MDR strains, three of which were from a genital source), CG60 (four ST60 strains from diverse clinical sources, half of which were MDR), and CG10451 (four ST145 strains from genital and digestive sources, half of which were MDR).

### Diversity of O-and K-antigens and comparison with K125 PCR detection

**Figure 6:**
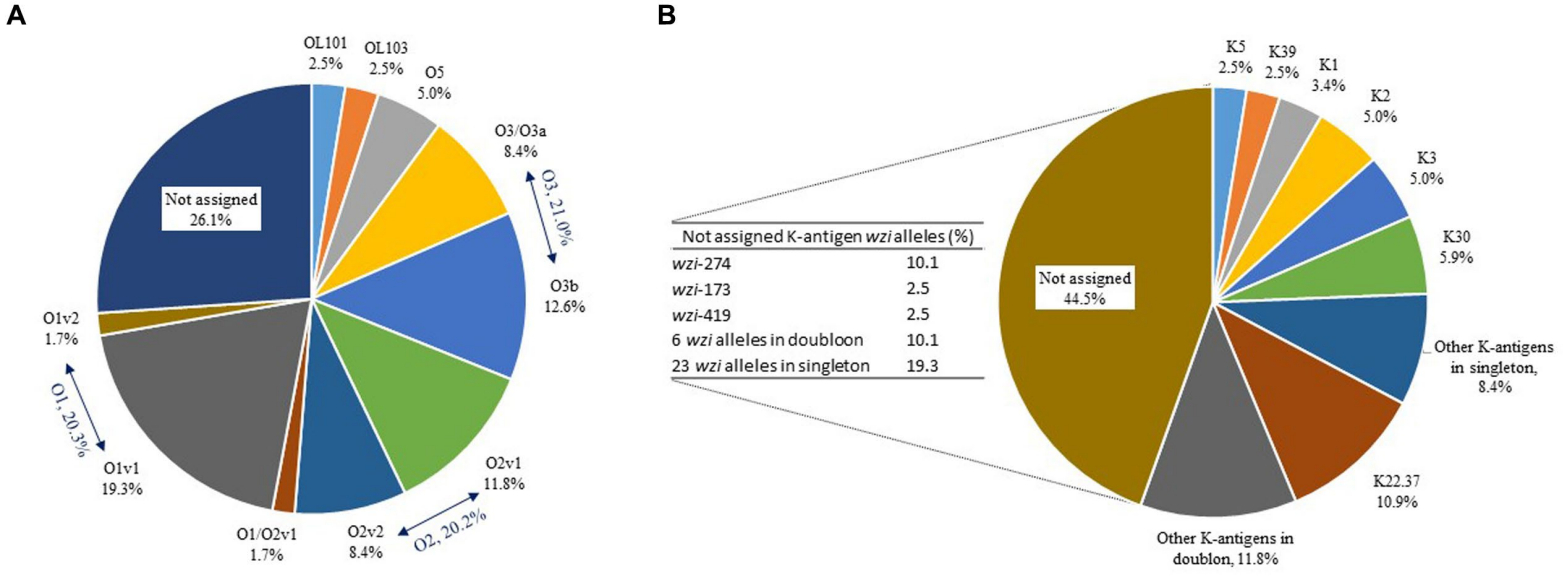
Distribution (%) of O-antigens **(A)** and K-antigens **(B)** in the 119 equine *K. pneumoniae* strains studied. Distribution (%) of *wzi* variants corresponding to the 44.5% of K-antigens not assigned is presented in the table to the left of the pie chart.

Ten different O-antigens were assigned for 88/119 genomes; O1v1 (*n* = 23, 19.3%) was the most prevalent followed by O3b (*n* = 15, 12.6%), O2v1 (*n* = 14, 11.8%), O2v2, O3/O3a (*n* = 10, 8.4% each), O5 (*n* = 6, 5.0%), OL101, OL103 (*n* = 3, 2.5% each), O1v2, and O1/O2v1 (*n* = 2, 1.7% each) (Figure 6A). Sixty-one *wzi* alleles were detected and 29 led to the assigning of 24 different K-antigens for 66/119 genomes, including the most prevalent K22.37 (*n* = 13, 10.9%), K30 (*n* = 7, 5.9%), K2, K3 (*n* = 6, 5.0% each), K1 (*n* = 4, 3.4%), and K5, K9 (*n* = 3, 2.5% each) (Figure 6B). Among the 32 *wzi* alleles that did not lead to the assigning of K-antigens for 53/119 genomes, *wzi*-274 (*n* = 12, 10.1%), *wzi*-173 (*n* = 3, 2.5%), and *wzi*-419 (*n* = 3, 2.5%) were the most prevalent (Figure 6B). Only 48 strains (40.3%) had a complete O:K serotype for a total of 24 different profiles, including the most prevalent O1v1:K22.37 (*n* = 8, 17.0%), O2v2:K30 (*n* = 5, 10.7%), and O1v1:K1 (*n* = 3, 6.4%) (Supplementary material). The O1v1:K22.37 profile was more frequent in MDR strains but this observation was not statistically significant (Fisher’s test, *p* = 0.0538).

The K1, K2, and K5 antigen assignments by WGS were compared with K125 PCR detection (Table 3). The results were consistent for 11/13 strains where a K1, K2 or K5 antigen had been assigned; the remaining two strains with a K2 antigen assigned by WGS resulted in a negative K125 PCR. Conversely, the K125 PCRs were positive for three strains carrying a *wzi*-181 variant (K1 PCR positive), an assigned K3 antigen (K2 PCR positive) and a *wzi*-482 variant (K5 PCR positive), respectively; however, the *wzi*-181, *wzi*-482 and K3 antigen assignment did not consistently show a positive K125 PCR result (Table 3).

**Table 3:** Comparison of assigned antigens K1, K2 and K5 by WGS and K125 PCR detection.

WGS		K125 PCR			
*wzi* variant	Assigned K-antigen	K1	K2	K5	Not detected
*wzi-12*	K1	4	0	0	0
*wzi-181*	Not assigned	1	0	0	1
*wzi-2*	K2	0	0	0	2
*wzi-72*	K2	0	4	0	0
*wzi-59*	K3	0	1	0	2
*wzi-5*	K5	0	0	3	0
*wzi-482*	Not assigned	0	0	1	1

### Distribution and identification of plasmids and virulence factors

Among the 119 strains studied, 29 plasmids were detected one to 34 times each, and 543 virulence genes and variants were detected one to 55 times each. Each strain showed the presence of zero to six plasmids with a median of one, and of eight to 60 virulence genes with a median of 14 (Supplementary material).

Plasmids are detailed in Table 4. The results revealed a high diversity of the incompatibility (Inc) groups. IncFIB(K) was detected in 28.6% (*n* = 34) of strains, including IncFIA(HI1) (*n* = 2), IncFIB(pNDM-Mar) (*n* = 2), and IncFIB(pKPHS1) (*n* = 1) co-detections. Twenty-two strains without IncFIB(K) harbored one or two other IncFI members, including IncFIA(HI1) (*n* = 7), IncFIB(K)(pCAV1099-114) (*n* = 12), IncFIB(pKPHS1) (*n* = 8), and IncFIB(pNDM-Mar) (*n* = 4). IncFII(K) was detected in 14.3% (*n* = 17) and three other IncFII members in 5.9% (*n* = 7). Other plasmids of an Inc. group were more infrequently detected, including five IncHI members, IncC, IncI1-I(Gamma), IncM1, IncN, IncQ1, IncR, and IncX3. Six Col plasmids, FIA(pBK30683), and RepB were also detected, the most frequent being Col(pHAD28) (*n* = 19, 16.0%) and ColRNAI (*n* = 9, 7.6%). Four Inc. plasmids and ColRNAI were statistically more frequent in MDR strains; IncHI1A and IncHI1B(R27) were more frequent in MDR strains but without statistical significance (Table 4).

**Table 4:** Plasmid identification.

Plasmid	All strains (*n* = 119)	MDR (*n* = 46)	not MDR (*n* = 73)	*p*-value^a^
Col (MG828)	1	0	1	1.0000^c^
Col (pHAD28)	19	7	12	0.8595^b^
Col156	1	0	1	1.0000^c^
Col440I	2	1	1	1.0000^c^
Col440II	4	2	2	0.6397^c^
ColRNAI	9	7	2	**0.0267** ^c^
IncC	8	7	1	**0.0053** ^c^
IncFIA (HI1)	9	7	2	**0.0267** ^c^
IncFIB (K)	34	16	18	0.2338^b^
IncFIB (K) (pCAV1099-114)	12	9	3	**0.0104** ^c^
IncFIB (pKPHS1)	9	6	3	0.0869^c^
IncFIB (pNDM-Mar)	6	4	2	0.2042^c^
IncFII	1	0	1	1.0000^c^
IncFII (K)	17	14	3	**0.0001** ^b^
IncFII (pHN7A8)	2	2	0	0.1474^c^
IncFII(pKP91)	4	0	4	0.1576^c^
IncHI1A	3	3	0	0.0554^c^
IncHI1B (pNDM-MAR)	7	4	3	0.4275^c^
IncHI1B (R27)	3	3	0	0.0554^c^
IncHI2	1	1	0	0.3866^c^
IncHI2A	1	1	0	0.3866^c^
IncI1-I (Gamma)	1	0	1	1.0000^c^
IncM1	1	1	0	0.3866^c^
IncN	6	4	2	0.2042^c^
IncQ1	1	1	0	0.3866^c^
IncR	9	1	8	0.1508^c^
IncX3	2	1	1	1.0000^c^
FIA (pBK30683)	2	1	1	1.0000^c^
RepB	1	1	0	0.3866^c^

a MDR strains were compared with strains that were not MDR.

b Pearson’s Chi-squared test.

c Fisher’s exact test.

Virulence factors are detailed in Table 5. As expected, the *mrk* type 3 fimbriae cluster was detected in 98.3% (*n* = 117) of strains, the remaining 1.7% (*n* = 2) carrying an incomplete cluster, *mrkACDFHIJ*. Aerobactin receptor *iutA* was detected in 95.0% (*n* = 115) but only one strain (strain 132) harbored both *iutA* and aerobactin cluster *iucABCD*. Yersiniabactin cluster *ybt-fyuA-irp* was detected in 31.1% (*n* = 37) and partially detected in 3.4% (*n* = 4), ferric uptake system *kfuABC* was detected in 28.6% (*n* = 34) and partially in 2.5% (*n* = 3), two-composent system *kvgAS* was detected in 22.7% (*n* = 27) and partially in 5.0% (*n* = 6), the *mce* microcin E492 cluster was detected in 10.1% (*n* = 12) and partially in one strain, salmochelin cluster *iro* was detected in 7.6% (*n* = 9), and mucoid phenotype regulators *rmpA* and *rmpA2* were detected in 7.6% (*n* = 9). Genes involved in the allantoin metabolism (*allABCDRS*, *arcC*, *fdrA*, *gcl*, *glxKR*, *hyi*, *ybbWY*, *ylbEF*) were co-detected with KP1_1364 and KP1_1371 in 10.9% (*n* = 13) of strains, while one strain harbored only *allB*. Colibactin cluster *clb* was detected in only two strains but was partially detected in 7.6% (eight strains did not harbor *clbK* and one strain harbored only *clbE*). Interestingly, yersiniabactin-and colibactin-encoding genes were statistically more frequent in MDR strains (Table 5).

**Table 5:** Virulence gene identification.

Gene	Virulence factor	All strains (*n* = 119)	MDR (*n* = 46)	Not MDR (*n* = 73)	*p*-value^a^
*mrkABCDFHIJ*	Type-3 fimbriae synthesis	117, 2 *mrkACDFHIJ*	44, 2 *mrkACDFHIJ*	73	1.0000^b^
*iutA*	Aerobactin synthesis	113	46	67	**0.0460** ^b^
*irp1, irp2*	Iron regulatory protein (yersiniabactin-encoding gene)	38, 3 *irp1*, 1 *irp2*	21, 1 *irp1*	17, 2 *irp1,* 1 *irp2*	**0.0232** ^b^
*ybtAEPQSTUX*	Yersiniabactin biosynthesis	38, 2 *ybtAQSTX*, 1 *ybtEPQT*	21, 1 *ybtAQSTX*	17, 1 *ybtAQSTX,* 1 *ybtEPQT*	**0.0148** ^b^
*fyuA*	Yersiniabactin uptake receptor / Iron uptake	40	22	18	**0.0092** ^b^
*kfuABC*	Ferric ionic-uptake system / Iron uptake	34, 1 *kfuB*, 2 *kfuC*	13	21, 1 *kfuB*, 2 *kfuC*	0.5963^b^
*kvgAS*	Two-component system KvgAS	27, 6 *kvgS*	9, 6 *kvgS*	18	0.3454^b^
*allABCDRS*	Allantoin metabolism	13, 1 *allB*	6, 1 allB	7	0.3534^b^
*arc*	Carbamoyl phosphate degradation	13	6	7	0.5564^b^
*fdrA*	Succinate-CoA ligase	13	6	7	0.5564^b^
*gcl, glxKR*	Glycolate degradation	13	6	7	0.5564^b^
*Hyi*	Glyoxylate metabolic process	13	6	7	0.5564^b^
*KP1_1364, KP1_1371*	Unknown function	13	6	7	0.5564^b^
*ybbWY*	Allantoin permease / Purine permease	13	6	7	0.5564^b^
*ylbEF*	Catabolic oxamate carbamoyltransferase	13	6	7	0.5564^b^
*mceABCDEGHIJ*	Microcin E492 (pore-forming bacteriocin) synthesis	12, 1 *mceHI*	7	5, 1 mceHI	0.2334^b^
*clbABCDEFGHIJKLMNOPQR*	Colibactin synthesis	2, 8 *clbABCDEFGHIJLMNOPQR*, 1 *clbE*	7 *clbABCDEFGHIJLMNOPQR, 1 clbE*	2, 1 *clbABCDEFGHIJLMNOPQR*	**0.0218** ^c^
*iroBCDN*	Salmochelin synthesis	9	5	4	0.3051^c^
*rmpA*, *rmpA2*	Regulator of mucoid phenotype A/A2	8 *rmpA*, 1 *rmpA2*	5	4	0.3051^c^
*iucABCD*	Aerobactin biosynthesis	1	1	0	0.3866^c^

a MDR strains were compared with strains that were not MDR.

b Pearson’s Chi-squared test.

c Fisher’s exact test.

The presence of a large virulence plasmid was sought by sequence homology with pLVPK (219,385 bp) and pK2044 (224,152 bp). The result suggests the presence of a plasmid of approximately 165 kb in strain 132 (75.2% coverage with pLVPK and 73.1% with pK2044), while all other strains showed coverage ranging from zero to 33.5% (Supplementary material).

**Figure 7:**
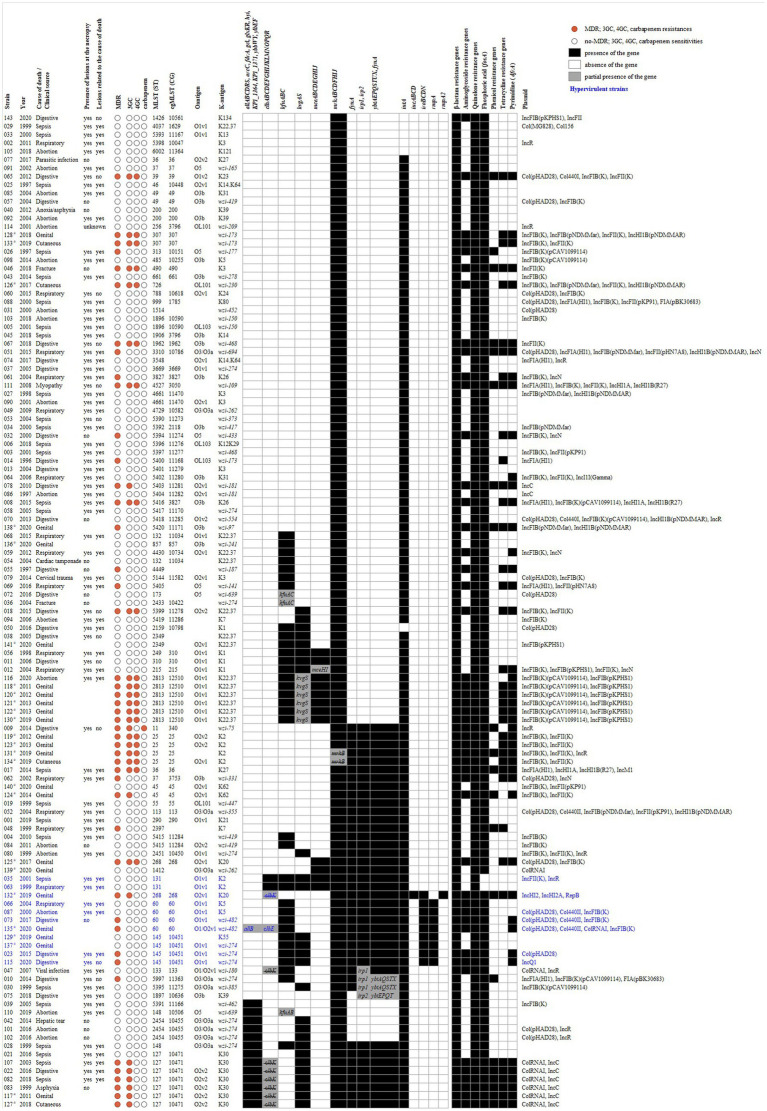
Heatmap of MDR, 3GC, 4GC, and carbapenem status, ST, O-antigen, K-antigen, virulence gene/cluster and antimicrobial resistance gene content of *K. pneumoniae* isolates (*n* = 119). The circular symbols represent MDR, 3GC, 4GC and carbapenem status as either MDR/resistance (red) or not MDR/sensitivity (white). The presence (black) or absence (white) of virulence genes/clusters and antimicrobial resistance genes is indicated. The partial presence of virulence clusters is highlighted in gray with a mention of the gene(s) present (gene not crossed out) or absent (gene crossed out). The antimicrobial resistance genes were grouped by antibiotic family and a positive result can concern more than one gene. Hypervirulent strains based on genotypic virulence profile are indicated in blue. Non-necropsy-associated strains (*n* = 25) are marked with an^a^.

Considering these findings, we highlighted 11 hypervirulent strains (9.2%) (indicated in blue in Figure 7); five of these were also MDR; including 3GC-resistant strain 132 (Figure 7). Nine strains harbored at least two to three biomarkers reported by Russo et al. (2018); in more detail, *mrk*, *iutA*, *ybt-fyuA-irp*, *iro*, and *rmpA* (eight strains) or *rmpA*2 (strain 132) loci were all detected, in addition to *iucABCD* and incomplete *clbABCDEFGHIJLMNOPQR* loci in K20-ST268 strain 132, *kfuABC* and *kvgAS* loci in *wzi 274*-ST145 strains 023, 115, and 137, and K55-ST145 strain 129, together with the *kfuABC* locus in K5-ST60 strains 066 and 087, and *wzi 482*-ST60 strains 073 and 135 (Figure 7); of note, a K125 PCR revealed K5 antigens in strain 135 (Table 3, Supplementary material). Five of these strains were necropsy-associated, and lesions related to the cause of death were reported at the necropsy for three of them (strains 23, 66, and 87) (Figure 7). Two additional necropsy-associated K2-ST131 strains—035 and 063—were also considered hypervirulent despite the absence of *peg-344*, *iroB*, *iucA*, *rmpA,* and *rpmA2* biomarkers reported by Russo et al. (2018) because they harbored *mrk*, *iutA*, *ybt-fyuA-irp*, *kfuABC, kvgAS*, *clb,* and *mce* loci; in both cases, lesions related to the cause of death were reported at the necropsy (Figure 7).

Other STs (e.g., ST25, ST2813) or K-antigens (e.g., K30, K22.37) may also be of interest due to the MDR status and the presence of several virulence genes (Figure 7).

## Discussion

The originality of our study is based on the high number of *K. pneumoniae* strains from both necropsy-associated and clinical isolates in horses. This panel of strains was isolated over a long collection period (1996 to 2020) covering the whole of France (i.e., national scale) and from various sample sources: necropsies, suspected bacterial infections (genital, wound, allantochorion, and umbilical artery samples) and contagious equine metritis analyses. This panel highlights the proportion of hypervirulent and/or MDR *K. pneumoniae* strains that have been circulating in French horses over the past few decades and ideally complements the limited number of publications dedicated to the detection of high-risk clonal lineages of *K. pneumoniae* in horses (e.g., Trigo da Roza et al., 2019; Loncaric et al., 2020; Shnaiderman-Torban et al., 2021).

The main finding of this study is the remarkable level of genomic diversity and the atypical panel among the 119 strains studied, suggesting a community acquisition of this pathogen that is frequently associated with equine clinical samples (Duchesne et al., 2019; Bourély et al., 2020; Léon et al., 2020). Thus, the most frequent populations were rarely described elsewhere with CG10451 (seven K30-ST127), CG12510 (six K22.37-ST2813), CG25 (four K2-ST25) composed of non-hypervirulent but MDR strains, and CG60 (four *wzi-482* or K5-ST60), CG10451 (four *wzi-274* or K55-ST145) composed of hypervirulent strains of which half were MDR. This finding does not confirm the conventional consideration of two individual populations associated with MDR strains (mainly belonging to CG258) and hypervirulent strains (mainly belonging to K1-CG23 and K2-CG25; note that four K2-CG25 strains were identified in our study, all were MDR but not hypervirulent) of *K. pneumoniae* found in humans (Holt et al., 2015; Russo and Marr, 2019; Wyres et al., 2020; Dong et al., 2022). In particular, we did not find any K1-ST23 strains while Lam et al.’s, 2018 study performed a comparative study of 97 genomes from both human and equine hypervirulent CG23 strains, including 15 equine K1-ST23 strains isolated from 1980 to 2004 in France. This epidemiological difference could be explained by the fact that their *K. pneumoniae* population was collected from genital samples, e.g., cervix, fetus, genital tract, mare metritis, stallion sperm (Lam et al., 2018), which was an origin that represented only 18% of the samples in our study. Furthermore, the only four K1 strains we identified were not of a genital origin. However, it is important to note that we found 11/119 (9.2%) hypervirulent strains based on virulence genes including the biomarkers described by Russo et al. (2018). Interestingly, seven of these strains were necropsy-associated. They could play a role in virulence and severe infections but did not cover all 65 horses (out of 94 necropsy-associated, 69.1%) of our panel where the lesions observed at necropsy were related to the cause of death (Supplementary material). Numerous genetic factors contribute to the ability of *K. pneumoniae* strains to cause severe diseases and probably most of them are still not known and/or not well enough known to affirm that they enhance severity, in particular in an unexplored host, like a horse. In our work, we revealed substantial allelic and gene content heterogeneity and sometimes partial coverage of well-described virulent plasmids such as pLVPK and pK2044 (e.g., MDR-hypervirulent strain 132). The extent and clinical impact of allelic and/or truncated virulence genes remain uncertain. In addition to this, we also need to determine by further studies the potential role of the other co-infecting bacteria found with *K. pneumoniae* (Supplementary material).

A highlight of our study is the description of five *K. pneumoniae* strains that are both MDR and hypervirulent, thus exacerbating the threat posed by very limited treatment options. The highly mosaic nature of *K. pneumoniae* plasmids creates the risk of MDR and virulence determinants converging within a single plasmid (Lam et al., 2019). However, this convergence was concentrated within a small number of STs comprising well-known hypervirulent (e.g., ST23, ST86, ST65) or MDR lineages (e.g., ST11, ST15, ST231, and ST147) (Lam et al., 2021). Geographically, the focal point for convergence appears to be Asia, where both MDR and hypervirulence are common (Wyres et al., 2020). Interestingly, four MDR-hypervirulent *K. pneumoniae* found in our horse panel belong to other STs (*wzi 482*-ST60 and *wzi 274*-ST145) that originate from France but had never been previously described. Only one strain in our panel—K20-ST268 strain 132—with 3GC resistance had already been described in a human healthcare surveillance system in China (Tang et al., 2020). The circulation of MDR-hypervirulent *K. pneumoniae* in horses in France is of concern from a One Health perspective, and requires greater awareness on a national scale.

MDR *K. pneumoniae* clones display much greater diversity and are known to be associated with a common cause of opportunistic infections in hospitalized patients. Highly-resistant lineages (including those resistant to 3GC and/or carbapenems) spread around the world rapidly. These MDR *K. pneumoniae* populations belong to CG258, CG15, CG29, CG37, CG147, and CG101 (Wyres et al., 2020). Here again, our work indicates sporadic or localized spread (confirmed by the cgMLST approach implemented) of rare and distinct STs that have rarely been described in the literature. Furthermore, we found a fairly high proportion of MDR strains out of the 119 studied (*n* = 46, 38.7%; including 31 strains (26.1%) resistant to at least one 3/4GC tested, mainly due to plasmid acquisition containing ESBL or AmpC genes). However, we also report the presence of several genotypes that are globally distributed and associated with MDR in humans (Dong et al., 2022), like the ST11 (CG258) carbapenemase producer, or ST307 (CG307) and K2-ST25, which concern, respectively, one, two and four strains among our panel of 3GC-MDR strains. A recent Israeli case control study on 3GC-resistant *Enterobacterales* infections in hospitalized horses and donkeys showed that the *Klebsiella* spp. were the most common 3GC-resistant *Enterobacterales* detected (Shnaiderman-Torban et al., 2021). Interestingly, we note a large increase in MDR between the 1996–2007 (nine MDR/48, 18.8%) and 2008–2020 (18 MDR/46, 39.1%) periods among the 94 necropsy-associated strains isolated. Seven hypervirulent strains out of 11 were isolated in the 2008–2020 period, which is also the case for all five MDR-hypervirulent strains studied.

A limitation of our work is that it is a retrospective study designed to include two distinct but complementary sources of *K. pneumoniae* strains of equine origin. The first one is based on nationwide diagnostic necropsy activity from 1996 to 2020, and the second one provides an antibiotic susceptibility profile and a selection of strains of genital origin isolated during the routine diagnostic activity of a regional veterinary laboratory, since *K. pneumoniae* is one of the pathogens sought before and during the breeding season among thoroughbreds, due to its propensity to cause metritis, infertility and abortion in mares (Léon et al., 2020).

Nevertheless, our analyses provide valuable insights and essential data to motivate enhanced public health surveillance among horses.

## Conclusion

In conclusion, our results clearly emphasize the importance of improving the surveillance of *K. pneumoniae* strains in routine equine diagnostic tests to detect high-risk MDR and/or hypervirulent strains. A better understanding of the epidemiological reservoirs of high-risk *K. pneumoniae* is needed to control their dissemination and provide essential data to public health surveillance bodies, both for humans and animals considering the One Health perspective. Furthermore, the circulation of these worrisome MDR-hypervirulent *K. pneumoniae* strains highlights the fact that they remain undetected by a simple diagnostic approach using K1, K2, and K5 serotypes as is implemented in the French horse-breeding sector. Further studies by genomic analyses are needed to propose better tools for improved epidemiological surveillance to estimate the burden of pathogenic *K. pneumoniae* strains in horses.

## Data availability statement

The datasets presented in this study can be found in online repositories. The names of the repository/repositories and accession number(s) can be found at: https://bigsdb.pasteur.fr/klebsiella/, (Klebsequi public projet id 15662-15676, 15689-15696, 21799-21801, 57541-57634). Genomes are also available on the NCBI database using the Bioproject number PRJNA1054041.

## Author contributions

FG: Data curation, Formal analysis, Investigation, Methodology, Software, Validation, Writing – original draft, Writing – review & editing, Resources, Visualization. CS: Investigation, Validation, Writing – review & editing, Resources. SC: Investigation, Writing – review & editing. NF: Investigation, Writing – review & editing, Resources. KM: Writing – review & editing, Resources. JT: Writing – review & editing, Resources. AL: Validation, Writing – review & editing, Resources. BL: Data curation, Writing – review & editing, Investigation, Software. SL: Investigation, Supervision, Validation, Writing – original draft, Writing – review & editing, Conceptualization, Methodology, Project administration, Visualization. SP: Conceptualization, Formal analysis, Funding acquisition, Investigation, Methodology, Supervision, Validation, Writing – original draft, Writing – review & editing, Project administration, Visualization.

## Footnotes

^1^http://www.eucast.org

^2^https://gitlab.pasteur.fr/GIPhy/fqCleanER

^3^https://bigsdb.pasteur.fr/klebsiella/

^5^https://github.com/katholt/Kleborate

^6^https://www.cgmlst.org/ncs/schema/2187931/
